# High performance bias-selectable three-color Short-wave/Mid-wave/Long-wave Infrared Photodetectors based on Type-II InAs/GaSb/AlSb superlattices

**DOI:** 10.1038/srep24144

**Published:** 2016-04-07

**Authors:** Anh Minh Hoang, Arash Dehzangi, Sourav Adhikary, Manijeh Razeghi

**Affiliations:** 1 Center for Quantum Devices, Department of Electrical Engineering and Computer Science, Northwestern University, Evanston, Illinois 60208, USA

## Abstract

We propose a new approach in device architecture to realize bias-selectable three-color shortwave-midwave-longwave infrared photodetectors based on InAs/GaSb/AlSb type-II superlattices. The effect of conduction band off-set and different doping levels between two absorption layers are employed to control the turn-on voltage for individual channels. The optimization of these parameters leads to a successful separation of operation regimes; we demonstrate experimentally three-color photodiodes without using additional terminal contacts. As the applied bias voltage varies, the photodiodes exhibit sequentially the behavior of three different colors, corresponding to the bandgap of three absorbers. Well defined cut-offs and high quantum efficiency in each channel are achieved. Such all-in-one devices also provide the versatility of working as single or dual-band photodetectors at high operating temperature. With this design, by retaining the simplicity in device fabrication, this demonstration opens the prospect for three-color infrared imaging.

Multispectral detection or simultaneous collection of signal from different infrared bands provides enhanced target discrimination and identification. The shortwave infrared (SWIR) wave band enables detecting reflected light which produces more intuitive and visible-like images. Meanwhile, longer wavelength regimes provide thermographic images. The mid-wave infrared (MWIR) detection is capable of detecting hot plumes while long-wave infrared (LWIR) as the peak of the radiation spectrum for room temperature objects can detect cooler bodies. Providing this new dimension of contrast, when coupling with powerful signal processing algorithms, multispectral detectors offer important advantages over single band detectors under a wide range of applications^1^,^2^. Third generation infrared imagers have considered multispectral detection as one of the key requirements^3^. While two-color detection (SW-MW, MW-LW, LW-LW) becomes increasingly popular^4^, there are several attempts in incorporating one more band to make three-color photodetectors^5^,^6^.

Recent trends in multispectral focal plane array (FPA) development has leaned toward integrating multicolor functionality into single pixels^4^. This approach removes the external parts such as beam splitters, filters, thus significantly reduces the size, weight, and power requirements. However, integrated pixel collocating multiband photodetectors face different kinds of challenges. The first major obstacle is the choice of material systems that have the ability to perform over a broad range of infrared spectrum. Due to the limit of available substrates, semiconductor materials are often limited to a particular detection window near the semiconductors’ bandgap. There are currently only a few material systems that are suitable for multispectral detection. The current state-of-the-art HgCdTe and quantum well infrared photodetectors (QWIPs) are commercially available for infrared dual-band detection^4^. However, HgCdTe technology is reported to suffer from high cost and low producibility yield while QWIPs have drawbacks of low quantum efficiency and lower operating temperature^4^. In that context, the 6.1 Angstroms family based Type-II InAs/GaSb/AlSb superlattices^7^ have proved to be an excellent alternative. Controlling the electronic structure by the layer thicknesses that are grown on GaSb substrate, Type-II superlattice (T2SL) offers the capability of tuning from SWIR^8^ to VLWIR^9^, covers the whole infrared spectrum. In recent years, T2SL material has not only matured in the improvement of single color detector performance but also witnessed a new phase of development for multi-band detection^10^ and imaging^11^. Two-color SWIR-MWIR^12^, MWIR-MWIR^13^, MWIR-LWIR^14^ and LWIR-LWIR^15^ focal plane arrays have been demonstrated with high performance.

The second major challenge resides in integrating a stack of absorbers into a single pixel. The incorporation of new channel usually needs additional terminal contacts, which will lead to more complicated fabrication process and thus, low yield. On the other hand, by defining additional metal contact, a portion of material must be etched away; the optical fill factor of stacked absorption channels in the pixels is therefore severely reduced^3^. This is particularly critical for applications which need high resolutions, namely detectors with smaller pitches. In order to mitigate these issues, two-terminal photodetectors with only a top and a common ground contact are more favorable. Besides providing a near unity optical fill factor, a single contact pixel has the advantage of simplicity on sharing the same fabrication process as single color or bias-selectable two-color arrays. In this matter, read out integrated circuits (ROICs) with one Indium bump could also be used for later Focal Plane Array (FPA) application.

In this article, we present our novel device design using only two terminals and report the experimental demonstration for the high performance triple-band T2SL-based SWIR/MWIR/LWIR photodetector based on this architecture. The core idea is to demonstrate devices that can perform sequentially as three individual single color photodetectors according to the magnitude variation of applied bias.

## Results and Discussion

**Figure 1:**
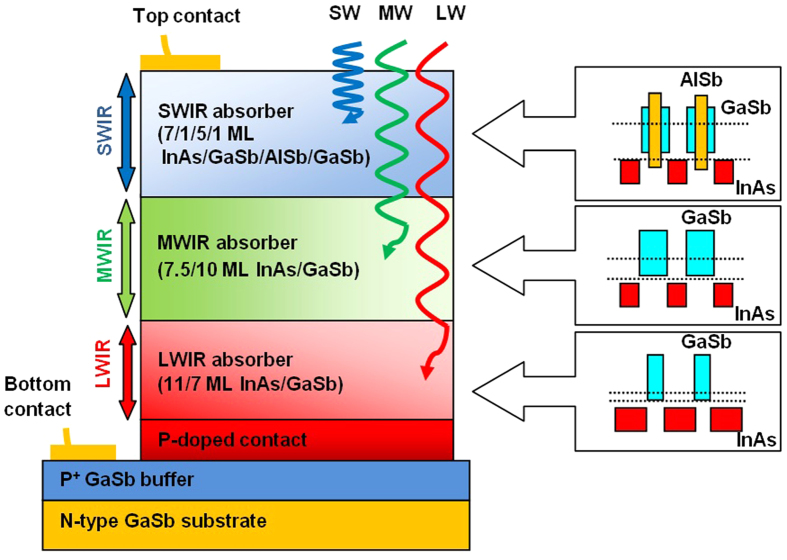
Schematic diagram of a triple-band SWIR-MWIR-LWIR photodiode structure with two terminal contacts and schematic band alignment of superlattices in three absorption layers. The colored rectangles in the insets represent the forbidden gap of component materials. Dotted lines represent the effective band gaps of superlattices.

The schematic diagram of top-side illumination device structure is shown in Fig. 1. Each layer absorbs radiation up to its cut-off and passes through longer wavelengths, which are then collected in subsequent layers. The SWIR channel is grown on the MWIR channel which is on top of the LWIR absorption layer. Using empirical tight binding model (ETBM)^16^, the design for SWIR absorber is 7.5/1/5/1 monolayers (MLs) of InAs/GaSb/AlSb/GaSb, aiming for 50% cut-off wavelength of 2.0 μm at 77 K. MWIR channel is designed with 7.5/10 MLs of InAs/GaSb with 50% cut-off wavelength of 4.5 μm, and the LWIR absorber is made of 11/7 MLs of InAs/GaSb, aiming for 9 μm cut-off at 77 K.

**Figure 2:**
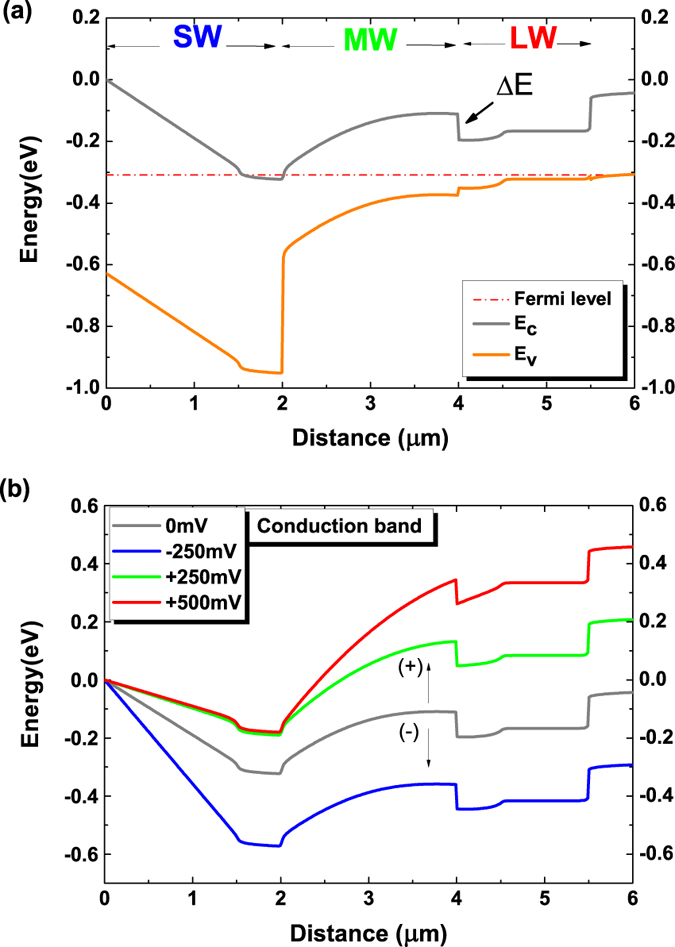
(**a**) Calculated band diagram showing the Fermi level, conduction band (E_c_) and valence band (E_v_) at equilibrium at zero bias. (**b**) Conduction band profile (E_c_) of designed structure at three operation bias regimes.

With only two contacts, the extraction of each color photocurrent therefore must be determined not only by the polarity of the applied bias but also by its magnitude. The design philosophy behind this device structure is to be able to control the distribution of electric potential along the device as function of applied bias, hence divide the applied bias into three separate intervals which will turn on the absorbers one after another. Previous reported triple-band HgCdTe with two terminal contacts used two spacer barriers to flank the intermediate layer^5^. This approach, however, is reported to be very challenging in controlling the spacer barriers to get well-defined cut-offs^17^. Here, we propose a novel approach in order to realize the device. Firstly, we separate SWIR from MWIR absorption by using a heavily n-doped SWIR layer between the SWIR and MWIR active regions. This will create two back-to-back photodiodes of SWIR and MWIR, operating at opposite bias polarities. Then, by using the blocking barrier created by the conduction band off-set ΔE (~126 meV) between the MWIR and LWIR regions, we can controllably determine the turn-on voltage for LWIR channel, thus separate LWIR from MWIR absorption. Bearing this in mind, the MWIR absorber is slightly p-doped. At the junction with MWIR absorber, a buffer layer of LWIR absorber is left undoped, followed by a p-doped LWIR absorber. Figure 2(a) shows the calculated band alignment and Fermi level across the entire device when no external bias is applied.

The operation principle of the designed device is depicted in the Fig. 2(b). At negative bias, SWIR channel is in reverse bias mode and the two other channels are in forward mode, the electrical current is governed by the SWIR diode (blue curve). At this mode, only SWIR radiation gets extracted. At positive bias, SWIR channel is now in forward while the MWIR and LWIR layers are in reverse bias. As soon as the applied bias is large enough to reduce the built-in voltage in the SWIR region, the MWIR absorption channel will be turned on (green curve). The dynamics of photocurrents in the dual-band back to back photodiodes was discussed in ref. 18. As a result, the SWIR absorber is intentionally left undoped in order to minimize the turn-on voltage for MWIR. At small positive bias, the carriers generated by LWIR radiation are blocked at the barrier; hence only MWIR radiation gets extracted. Only after a large enough bias is applied, when the depletion region spreads all over the MWIR absorber and gets into the undoped LWIR layer, the carriers generated in the LWIR channel are able to cross the barrier and contribute to the photocurrent (red curve). With this technique, the cascade of more waveband detection is possible in principle. The challenges here reside essentially in controlling the MWIR-LWIR junction to ensure that the turn-on voltage for LWIR channel is always larger than the turn-on voltage for MWIR channel.

There are three main factors that directly affect the turn-on voltage for LWIR channel: the conduction band off-set, the discrepancy in doping levels that will determine the depletion region and the thickness of MWIR channel. Since the band off-set ΔE is fixed and the layer thickness is kept reasonably thick to achieve high optical response, we focus our efforts on the investigation of doping levels. In this work, we study the MWIR absorber doping level to tune the conduction band barrier, hence achieve the desired behavior with three operation regimes having three separate cut-offs.

For experimental demonstration, triple-band SW-MW-LW photodiodes are designed to consist of 1.5 μm thick undoped active region in SWIR, 0.5 μm thick n-doped SWIR (n ~ 10^18^ cm^−3^), 2.0 μm thick MWIR active region, 0.5 μm thick undoped followed by 1.0 μm thick p-doped LWIR active region (p ~ 10^16^ cm^−3^) and 0.5 μm thick bottom p-contact (p ~ 10^18^ cm^−3^). The total thickness of the device structure is 6 μm. The effect of p-doping MWIR absorber on optical behavior is studied via five samples (denoted as letters A, B, C, D, E) with varying Beryllium dopant cell temperature (830, 800, 780, 750 and 700 °C), corresponding to a range between p ~ 10^14^–10^16^ (cm^−3^)^19^.

**Figure 3:**
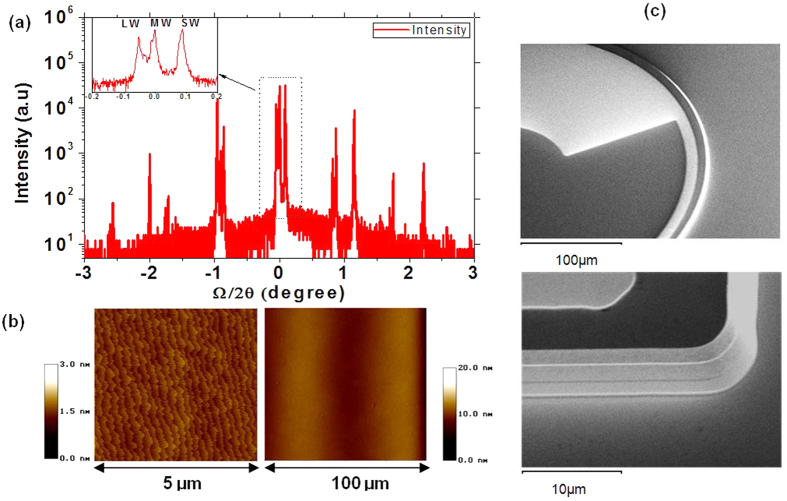
(**a**) HR XRD rocking curve (inset: zero order peaks of SL and substrate). (**b**) AFM of 5 × 5 μm^2^ (rms roughness = 1.0 Å) and 100 × 100 μm^2^ area; (**c**) SEM pictures of processed single element devices.

After the growth, the samples were structurally characterized using High resolution X-ray diffraction (HR XRD) and Atomic force microscopy (AFM) as shown in Fig. 3(a,b). The satellite peaks in the XRD rocking curves show the thicknesses of 60, 59 and 48 Å for each period of LWIR, MWIR and SWIR active regions, respectively. The AFM shows a standard morphology with roughness mean square of 1.0 Å over a 5 × 5 μm^2^ area and a smooth surface over a large area 100 × 100 μm^2^.

The grown materials were then processed into square and circular mesa-isolated single element diodes with sizes ranging from 100 to 400 μm in diameter. Pictures of the processed circular optical diode top side and square diode side wall under scanning electron microscope (SEM) are shown in Fig. 3(c). After the processing, the samples were wire-bonded onto a leadless ceramic chip carrier (LCCC) and loaded into a cryostat for optical and electrical characterizations.

**Figure 4:**
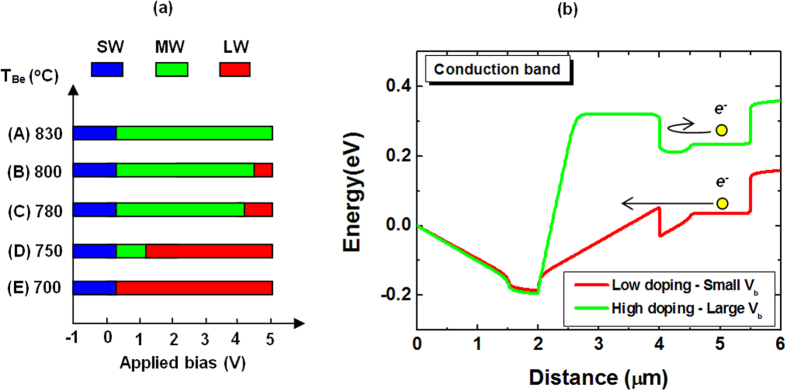
(**a**) Cut-off wavelength as function of applied bias for samples with different MWIR absorber doping levels. (**b**) Schematic conduction band of samples with high doped (green) and low doped (red) MWIR absorber. The excited electrons from LWIR absorber are blocked by MWIR channel even at high bias in the former case, and pass freely at low bias in the latter.

Figure 4(a) shows the cut-off wavelength variation of the devices as function of applied bias. There are two scenarios in which the devices behave as two-color devices. At one end, in sample A with highly doped MWIR layer (Be = 830 °C), the photocurrent from the LWIR absorption channel is entirely blocked, even at high positive bias voltage as represented by the green curve in Fig. 4(b). Most of the bias drops at the SWIR-MWIR junction and has little effect on LWIR-MWIR junction. The device is a two-color SWIR-MWIR photodetector. At the opposite end, sample E with low p-doped MWIR absorber does not have a clear cut-off for MWIR. Indeed, as can be seen from the red curve in Fig. 4(b), since the MWIR channel is soon depleted, the LWIR channel is turned on at small positive bias as soon as the SWIR is off, it overlaps the MWIR response. The device actually performs as a two-color SWIR-LWIR photodetector. The desired behavior of a three-color photodiode only appears in three samples B, C, and D which have moderate doping levels. Samples B and C with Be at 800 and 780 °C exhibit a LWIR response at pretty high bias, starting around 4.5 and 4 volts, respectively. Even though these two samples have three-color behavior, the high turn-on voltage for LWIR is not desirable since it results in high noises.

**Figure 5:**
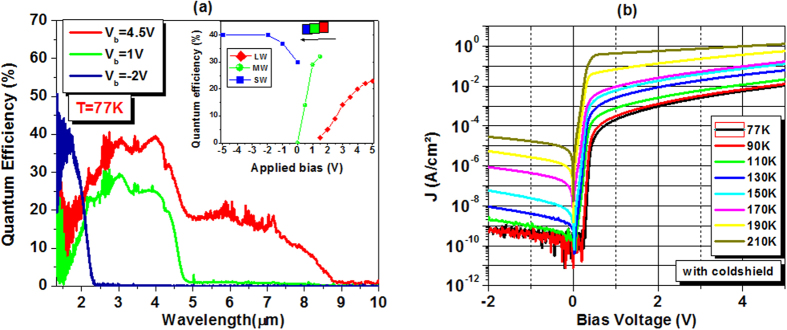
(**a**) Quantum efficiency spectrum of the photodiode at 77 K as function of applied bias. Inset: QE level at the wavelength of interest. (**b**) Current-voltage characteristics as function of operating temperature.

The optimal doping level is in sample D with Be temperature at 750 °C. Three quantum efficiency (QE) spectra at three different applied biases (V_b_ = −2, 1 and 4.5 V) are shown in Fig. 5(a). Going from negative to positive bias, the device exhibits a well defined cut-off wavelength shifting from SWIR to MWIR and then, LWIR. Indeed, only SWIR signal is collected at zero and negative bias, while MWIR response shows up from 500 mV, and only beyond 1500 mV, we can start collecting LWIR response. At 77 K, the device’s 50% cut-off wavelength is 2.0 μm for SWIR, 4.6 μm for MWIR and 8 μm for LWIR. The QE in the wavelength of interest is depicted in the inset of Fig. 5(a). In negative bias, SWIR QE saturates at about −2 V and reaches 40%, corresponding to the responsivity of 0.54 A/W at its peak (~1.7 μm). In positive bias, where the device’s cut-off is still MWIR, the quantum efficiency is 25% at peak responsivity of 0.8 A/W (~4.0 μm) at 1 V. LWIR QE increases steadily from 1500 mV and saturates at around 4.5 V, achieves a value of 19%, equivalent to responsivity of 1 A/W at its peak (~7.2 μm). At this bias, QE at MWIR is also increased to 40% (responsivity = 1.3 A/W). The spectral response is the overlap of photocurrents contributed from both LWIR and MWIR absorbers. Simple signal processing by the subtraction of MWIR response should be able to give the signal response from LWIR absorber. The level of QE can be further improved by increasing the thickness of the absorption layer. However, it should be noticed that more optimization is required to reduce the bias dependency for LWIR response in order to make it suitable for FPA imaging application. Fine tuning of Beryllium temperature or a graded doping profile could be used for further optimization to reduce the applied bias.

Shown in Fig. 5(b) is the device’s electrical performance measured at different temperatures ranging from 77 to 210 K. At 77 K, the dark current density at negative bias, where SWIR signal is extracted, reaches our system floor level, with value at the range below 1.0 × 10^−9 ^A/cm^2^ at −2 V. For MWIR operation regime, the dark current density is 2.1 × 10^−4 ^A/cm^2^ at 1 V. And at 4.5 V, where the LWIR is fully collected, the dark current density is 7.6 × 10^−3 ^A/cm^2^. At 150 K, the dark current density for SWIR, MWIR and LWIR operation is 5.8 × 10^−8^, 5.9 × 10^−3^ and 9.8 × 10^−2 ^A/cm^2^, respectively. It is worth mentioning that with the capability of choosing cut-off wavelengths, the device can still be functioning as two-color (SWIR-MWIR) or single color (SWIR) detector at high operating temperature.

**Figure 6:**
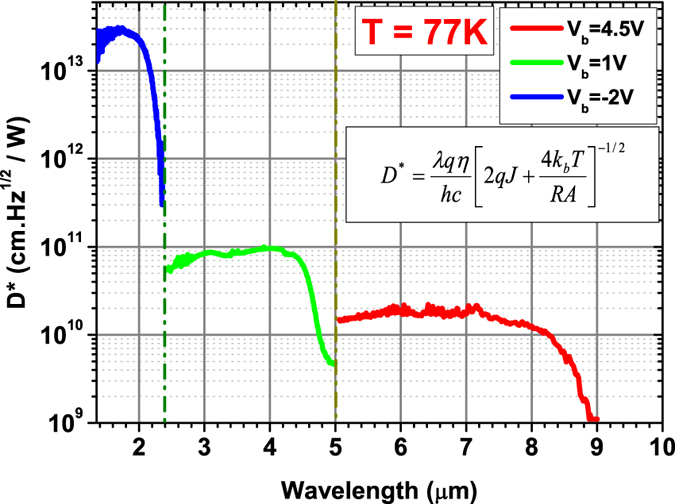
Detectivities of three channels at the wavelengths of interest at 77 K. The SWIR detection operates at −2 V. The MWIR detection operates at 1 V and LWIR detection operates at 4.5 V positive bias voltages. The detectivity calculation uses the equation in the inset, where λ is wavelength, n is QE, J is dark current density, RA is differential resistance-area product, h, c, and k_b_ are basic constants.

Shown in Fig. 6 are the calculated shot noise limited detectivity D* of the device in its three operation modes at 77 K based on the measured quantum efficiency, the dark current and the resistance-area product. The device operates as a SWIR, MWIR and LWIR photodetector at bias of −2, 1 and 4.5 V and provide a D* of 3.0 × 10^13^, 1.0 × 10^11^ and 2.0 × 10^10^ cm. 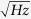/W at peak responsivity (λ = 1.7, 4.0 and 7.2 μm). Compared to the previously reported results for three-terminal three-color photodiodes^6^, the device performance is one to three orders of magnitude better while retaining the simplicity in device fabrication. The high performance of the SWIR channel grown on top of MWIR and LWIR channels confirms the high quality of T2SL material for complex thick structure.

## Conclusion

In summary, the novel device architecture for the realization of triple-band SWIR-MWIR-LWIR photodetectors with two terminals has been proposed and discussed. We reported the concept demonstration of the bias-selectable triple-band SWIR-MWIR-LWIR photodetectors based on the Type-II InAs/GaSb/AlSb superlattices. By studying and finding an appropriate doping level for the MWIR absorber, the photodiode sequentially exhibited the behavior of three photodiodes of different colors, corresponding to the bandgap of three absorbers. At 77 K, the SWIR channel achieved a quantum efficiency of 40% at peak responsivity, giving a detectivity of 3.0 × 10^13^ Jones. The MWIR channel with p-doped active region had its range of operation from 500 to 1500 mV and exhibited a detectivity of 1.0 × 10^11^ Jones. The LWIR channel operated with bias beyond 1500 mV and reached its saturated QE of 19% at 4.5 V, providing a detectivity of 2.0 × 10^10^ Jones. This work has proposed a novel approach in making multispectral infrared imagers and for the first time, successfully demonstrated bias-selectable three-color SW-MW-LW photodiodes with two terminal contacts using InAs/GaSb/AlSb Type-II superlattice.

## Method

### Growth and fabrication

The samples were grown on tellurium (Te)-doped (001) GaSb (n) substrates using the Molecular Beam Epitaxy (MBE) equipped with group III SUMO® cells and group V valved crackers. Silicon and beryllium were introduced in InAs and GaSb or AlSb as n-type and p-type dopants, respectively. The *in situ* Reflection high energy electron diffraction (RHEED) was used to monitor the growth process.

The processing of single element photodiodes can be separated into two main steps as mesa isolation and metallization. After the sample preparation with regular cleaning and degreasing, standard photolithography was performed to make the photoresist (PR) pattern on top of the samples. The pattern was transferred onto the material to form single element photodiodes by inductively coupled plasma -reactive ion etching (ICP-RIE) (Oxford PlasmaLab System 100), using BCl_3_/Ar plasma chemistry and citric-acid based wet etching. Using high density plasma etchers such as the ICP-RIE enabled us to benefit from low process pressures, which was set at 2 mTorr in our case. For dry etching procedure, the table temperature, ratios between BCl_3_ and Ar flow rates, and RIE/ICP powers were optimized accordingly. After dry etching, citric acid base isotropic wet etching was applied in order to provide more uniform etching, remove residues of the dry etching and regenerate smoother sidewalls. After etching procedure, solvent-based cleaning was performed on all samples to remove the photoresist residues and byproducts from the etching steps, and to ensure sidewall cleanliness. This cleaning procedure is very important for the devices, since residues and byproducts can easily cause surface leakage current, which may degrade the photodiode performance. Then, top and bottom metal contacts (Ti/Au) deposition was performed by electron beam metal evaporation, following by lift-off processing. Careful selection of metal for top and bottom contacts was required so an Ohmic contact must be formed to prevent potential barriers between the semiconductor and metal junction. After lift-off, another cleaning procedure was performed on samples. The samples were left unpassivated but special attention was paid in order to minimize the surface leakage during all fabrication procedure.

### Device testing

The processed photodiodes were mounted onto a 68-pin leadless chip carrier (LCC) with indium for electrical and optical characterization. The connections from the top and bottom contact of the sample to the bond pads of the LCC were made with a resistance-heated thermo compression wire bonder. After bonding, the samples were loaded into a Jenis STVP-100 two chamber liquid helium cryostat which had the capability to measure from 4 K to 300 K, and connected to different equipments using switching matrix for various type of measurement. The measurement of optical response consisted of two parts: measurement of relative spectral response and measurement of the calibrated blackbody integrated response. The relative spectral response of a detector is measured by a Bruker IFS 66/v Fourier transform infrared spectroscopy (FTIR) system.

## Additional Information

**How to cite this article**: Hoang, A. M. *et al*. High performance bias-selectable three-color Short-wave/Mid-wave/Long-wave Infrared Photodetectors based on Type-II InAs/GaSb/AlSb superlattices. *Sci. Rep.* **6**, 24144; doi: 10.1038/srep24144 (2016).
